# Using the WHO individual near miss case review (NMCR) cycle to improve quality of emergency obstetric care and maternal outcome in Keren hospital, Eritrea: an interrupted time series analysis

**DOI:** 10.1186/s12884-024-06482-3

**Published:** 2024-04-11

**Authors:** Henos Kiflom Zewde

**Affiliations:** Department of Family and Community Health, Ministry of Health Anseba Region Branch, Keren, Anseba Eritrea

**Keywords:** WHO near miss case review, Quality improvement, Emergency obstetric care, Eritrea

## Abstract

**Background:**

In 2016, the WHO regional office for Europe prepared a manual for conducting routine facility based individual near miss case review cycle. This study evaluates the effectiveness of the individual near miss case review (NMCR) cycle in improving quality of emergency obstetric care and maternal outcome in Keren hospital.

**Methods:**

An interrupted time series design was used to achieve the objectives of this study. Monthly data on women with potentially life-threatening conditions (PLTCs) admitted between April 2018 and October 2022 (i.e. 33 months pre-implementation and 22 months post-implementation) were collected from medical records. Segmented regression analysis was used to assess the intervention’s effect on three process and outcome measures, namely, SMO, delayed care, and substandard care. The intervention was expected a priori to show immediate improvements without time-lag followed by gradual increment in slope. Segmented regression analyses were performed using the “itsa’ command in STATA.

**Results:**

During the entire study period, 4365 women with potentially life threatening conditions were identified. There was a significant reduction in the post-implementation period in the proportion of mothers with PLTC who experienced SMO (− 8.86; *p* <  0.001), delayed care (− 8.76; *p* <  0.001) and substandard care (− 5.58; *p* <  0.001) compared to pre-implementation period. Results from the segmented regression analysis revealed that the percentage of women with SMO showed a significant 4.75% (95% CI: − 6.95 to − 2.54, *p* <  0.001) reduction in level followed by 0.28 percentage points monthly (95% CI: − 0.37 to − 0.14, *p* <  0.001) drop in trend. Similarly, a significant drop of 3.50% (95% CI: − 4.74 to − 2.26, *p* <  0.001) in the level of substandard care along with a significant decrease of 0.21 percentage points (95% CI: − 0.28 to − 0.14, *p* < 0.001) in the slope of the regression line was observed. The proportion of women who received delayed care also showed a significant 7% (95% CI: − 9.28 to − 4.68, *p* < 0.001) reduction in post-implementation level without significant change in slope.

**Conclusions:**

Our findings suggest that the WHO individual NMCR cycle was associated with substantial improvements in quality of emergency obstetric care and maternal outcome. The intervention also bears a great potential for scaling-up following the guidance provided in the WHO NMCR manual.

**Supplementary Information:**

The online version contains supplementary material available at 10.1186/s12884-024-06482-3.

## Introduction

During the past three decades, the proportion of institutional delivery increased in most low- and middle-income countries [1]. However, increased coverage of delivery and perinatal care do not necessarily equate to better maternal and newborn outcomes [2]. Unless accompanied by evidence-based emergency obstetric care, the expansion of essential obstetric interventions will not be enough to improve maternal outcome [3]. The majority of maternal deaths occur during labour or in the early postpartum period [4] and are largely related to poor quality of care [5]. According to estimates from a recent multi-country analysis conducted in 81 low- and middle-income countries, the global sum of maternal deaths could have been reduced by 28% had there been high quality obstetric care provided in well-functioning health systems [5]. Hence, quality of care has gained increasing international recognition as an integral and critical aspect of the unfulfilled global health agenda of ending preventable maternal mortality [6].

The concept of obstetric audit and review has long been recommended as a quality improvement practice in tertiary facilities of developing countries [7]. Obstetric review is a cyclic and systematic analysis of obstetric care practices against predefined standards to optimize clinical performance [8]. The review cycle begins by establishing criteria of good practice, measuring current practice, providing feedback and setting targets, implementing indicated changes in practice, and finally re-evaluating practice and feedback before starting the review cycle all over again [8]. Findings from previously conducted systematic reviews indicated that obstetric reviews might have small to moderate effect in improving professional practice [9–11]. Since audits are more likely to have a significant effect when the baseline quality of care is poor [10], they are more likely to be useful in resource-constrained settings of developing countries.

Criterion based audit and individual NMCR are the two most commonly used methods of review in obstetrics [11]. Individual near miss case review (NMCR) is a method of audit where structured meetings to review the management of individual near miss cases are regularly conducted using a checklist of standard processes of care [7]. It is different in principle from criterion-based audit although the two terms have been used interchangeably by some authors [11]. They are similar in that all methods of obstetric review try to make improvements in the quality of emergency obstetric care in accordance with evidence-based standards of care. However, unlike criterion-based audits where limitations in care are discussed at the aggregate level, individual near-miss reviews emphasize on active involvement of the care providers through meetings held on regular bases, usually once a month. Its main advantages are that it is inexpensive, simple and requires little external assistance or data analysis skills [8].

Previous studies examining the effectiveness of audits in improving obstetric practice have produced inconsistent findings that range from a seemingly negative effect to a strongly positive effect [9]. In addition, most studies focused on evaluating obstetric reviews that address specific obstetric complications such as obstructed labour [12, 13], obstetric haemorrhage [14, 15], uterine rupture [16] and hypertensive disorders of pregnancy [17, 18]. Moreover, the majority of them evaluated quality improvement processes that rely on explicit and pre-specified criterion-based standards, giving little regard for pragmatic and empirical assessment of care.

In 2016, the WHO regional office for Europe prepared the first comprehensive manual with detailed guidance and instruction on how to conduct a routine facility-based individual near miss case review [19]. The manual takes a bottom-up approach and addresses the factors that have been shown to act as barriers to effective and sustained functioning of NMCR [20]. It urges exclusive discussion of near-miss cases during review sessions, as near miss cases are more abundant than maternal deaths [21] and discussing them is less likely to instigate blame compared to discussing maternal deaths [22]. According to the manual, midlevel staff such as midwives and nurses take a central place in the individual NMCR sessions, as obstetric reviews tend to be more effective when conducted by colleagues rather than external reviewers and researchers [10]. In line with the principle that women’s actual experience of care is as important as the effectiveness of care provided in facilities [6], the manual also contains detailed instructions on how to interview women and how to use the collected information to improve the quality of care from the mothers’ perspective [19].

A previous study at Keren hospital investigated the quality of emergency obstetric care and its association with maternal outcome [23]. The study identified significant delays and deficiencies in quality of care, where only 59.4% of women with ruptured uterus underwent laparotomy within 3 hours of hospital stay, and 69% of women having cesarean sections did not receive prophylactic antibiotics. Following this baseline study [23], individual NMCR cycle was recommended as a quality improvement intervention in the hospital. The review cycle was implemented in compliance with the instructions and recommendations elucidated in the WHO NMCR manual. This study evaluates the effectiveness of introducing the individual NMCR as a cyclic process to improve the quality of emergency obstetric care and maternal outcome in the emergency obstetric care facility of Keren hospital.

## Methods

### Study design

We used an interrupted time series design to evaluate the association between the implementation of the individual NMCR cycle and improvements in the quality of emergency obstetric care and maternal outcome.

### Study setting and context

This study was conducted in Keren Regional Referral Hospital. Located in the town of Keren, the capital of Anseba Province, the hospital is the largest in the province. It is the only referral hospital providing comprehensive and emergency obstetric care for women with maternal complications in the entire province. It is a public hospital that serves women referred by 3 community hospitals, 8 health centers, and 35 health stations in surrounding districts. In total, the hospital accommodates 80 beds in the maternity ward. It provides reproductive health, child health and emergency obstetric care services free of charge. On average, the hospital serves approximately 549,000 residents of the province. Twenty-one health assistants, seven nurse midwives, two senior obstetricians, and two anesthetists operate the maternity unit of the hospital. Even though conducting maternal death review is a routine practice in the hospital, staff members had not had any experience related to near-miss case review prior to the commencement of the individual NMCR cycle in the hospital.

### The intervention

The individual NMCR cycle was implemented as a routine quality improvement practice in Keren Hospital starting in January 2021. The review cycle was introduced following a study conducted in the hospital to identify deficiencies in the quality and timeliness of emergency obstetric care [23]. Training was given to all members of the hospital who participate in the provision of emergency obstetric care before the introduction of the review cycle. The training was 15–18 hours long and was given over a period of 6 days. The principal researcher along with two regional authorities of maternal and child health were responsible for the training. A clear and detailed explanation was given regarding the purpose of the review. Participants were also trained on how to conduct a quality individual NMCR and they practiced a simulation exercise at the end of the training. The review cycle is conducted in accordance with the recommendations given in the NMCR manual developed by the WHO regional office for Europe [19]. Review sessions are held monthly and each session usually lasts 1 hour to 1 hour and a half. Every session begins by revising the implementation status of the recommendations from previous meetings. Then, the facilitator presents a case summary of the case(s) selected for the day (usually one or two cases are selected per session), and the participants provide recommendations after discussing and analyzing the quality of emergency obstetric care received by the case(s) selected for discussion. External evaluators visit the hospital once every 6 months to evaluate the quality of the individual NMCR cycle using the checklist provided in the manual [19], and provide instant feedback. A detailed description of the intervention has been provided in the supplementary material using the template for intervention description and replication (TIDieR) checklist (Additional file 4) [24].

### Study period

Since the individual NMCR sessions are conducted every month and new quality improvement recommendations are proposed monthly, we opted to collect monthly data on the outcome variables for the purpose of this study. The entire study period for this study extended for 55 months (33 months pre-implementation and 22 months post-implementation). For the pre-implementation period, monthly data were collected on the outcome variables beginning from April 2018 until December 2020. This duration of time is believed to provide us with enough data points to understand the trend in the pre-implementation period and to make a better approximation of the counterfactual. The post-implementation period, on the other hand, comprises monthly data points starting from January 2021 (the month when the individual NMCR cycle started) to October 2022. The post-implementation period was set a priori based on the author’s assumption that a period of 2 years will be enough for the individual NMCR cycle to show its ultimate impact on quality of emergency obstetric care and maternal outcome.

### Data source and outcome variables

Data for this study were collected from patient registers and medical records of the hospital. All women who were in labor, delivered or aborted, or within 42 days postpartum, and admitted to maternity or emergency wards of this hospital were the source population for this study. Only women who experienced a potentially life-threatening condition (i.e. severe postpartum hemorrhage, severe pre-eclampsia, eclampsia, sepsis/severe systemic infection, and ruptured uterus) as per the criteria provided in the WHO maternal near miss (MNM) tool [25] were considered eligible for this study. We decided to consider only women with PLTC to account for the month-to-month variation in the number of women who needed immediate and critical intervention. Two senior obstetricians identified all women who experienced PLTC from delivery wards, obstetric wards, and emergency wards of the hospital and collected data on the selected outcome variables.

We used the Donabedian model of quality assessment to evaluate the impact of the individual NMCR cycle in improving the quality of obstetric care and maternal outcome [26]. Donabedian proposes gathering information on three characteristics of care for making inferences about the quality of care, namely structure, process, and outcome of care. In this study, we collected data on variables related to timeliness and standard of care to evaluate the impact of the individual NMCR cycle on process of care. We also collected data related to maternal outcome to evaluate the intervention’s effect on outcome of care. However, we were not able to collect data related to structural aspects of care as variables related to structural elements of care are hardly available as part of the routinely collected information in the hospital. Accordingly, data on the following categories of variables were collected from the medical records of each eligible woman using a structured checklist:

- *Variables related to standards of care*: whether a woman with PLTC received substandard care based on the list of standard process of care indicators mentioned in the WHO MNM tool [25].
- *Variables related to timeliness of care*: whether a woman with PLTC experienced delay in receiving any of the critical medical interventions stated in the framework proposed by Edson et al. [27].
- *Maternal outcome variable*: whether a woman with PLTC experienced severe maternal outcome (SMO) as defined in the WHO MNM tool [25].

### Aggregate outcome measures

Using the categories of variables related to timeliness of care, standard of care and SMO, we created three aggregate outcome measures. These aggregate outcome measures were used as a proxy to measure the improvements made in the hospital with regard to the process of care and maternal outcome. They were calculated on a monthly basis starting from the first month of the pre-implementation period. The first outcome measure is the proportion of women with PLTC who experienced severe maternal outcome (SMO), which is defined as the total number of SMO cases in a given month (numerator) divided by the total number of women with PLTC identified in that particular month (denominator). The second assesses the fraction of all women with PLTC (denominator) who experienced substandard care (numerator). Since our study was underpowered to fit a separate regression model for each substandard care indicator, we decided to calculate the aggregate coverage of the process indicators on a monthly basis. Finally, the third outcome measure is the proportion of women with PLTC admitted to the hospital (denominator) who encountered delays in receiving critical interventions (numerator). Operational definitions of all outcome variables and the methods used to calculate aggregate outcome measures have been provided as supplementary material (Additional file 2).

### Data collection process and data quality assurance

The entire data for this study were collected over 2 months period between January, 52,023 and March, 092023.Two senior obstetricians conducted screening in all relevant medical registers of the hospital to find cases who met the criteria of PLTC based on the MNM tool proposed by the WHO. Once all eligible women were identified, the data collectors retrieved the patient card of each qualified woman to fetch more detailed information. Additional data sources, such as laboratory registers (to gather data on the types of laboratory investigations ordered, when they were ordered and the results) and individual patient records (to get detailed information regarding the physician’s impression of the case), were also referred to when necessary. The obstetricians who collected data for this study were intentionally blinded to the starting date of the intervention to mitigate the occurrence of observer bias during data collection (Additional file 5). Although most outcome variables used in this study are objective, we were aware that variables related to timeliness of care might heavily depend on the subjective judgement of the data collectors. Hence, both data collectors assessed 10% of all eligible women at the beginning of the data collection process to ensure that there was substantial agreement between the two reviewers. Cohen’s kappa statistic was then used to evaluate the inter-observer agreement, and its value was above 0.9, indicating a near perfect concordance between the two data collectors. There was no difference in the source or methods of data collection between the pre-implementation and post-implementation periods. The hospital’s data handling and management practice was excellent, and therefore, data collected from medical registers and patient cards were complete without substantial missing information.

## Statistical analysis

Interrupted time series analysis was employed to assess the effectiveness of the individual NMCR cycle. The intervention was anticipated a priori to follow a step and slope change model. A step change model was hypothesized since interventions intended for quality improvement, which are proposed in the monthly individual NMCR sessions, are expected to be implemented immediately in the following month, and thus lead to instantaneous improvements in quality of emergency obstetric care and maternal outcome. A slope change model, on the other hand, was anticipated based on the assumption that new recommendations generated in the subsequent months will continue to make gradual improvements in the quality of emergency obstetric care and maternal outcome.

All statistical analyses were performed using Stata 14. The proportion of each outcome measure was calculated for the pre and post-implementation periods using summary statistics. Equality of the pre and post-implementation proportions was then assessed using the “*prtest*” command. All interrupted time series analyses were performed using the user-written “*itsa*” command in Stata [28]. The presence of autocorrelation was then assessed using the “*actest*” command. Whenever autocorrelation was identified, the model was re-estimated, specifying the lag that correctly accounted for the autocorrelation. Similarly, the augmented Dickey-Fuller test was used to check whether the time series data were stationary. The differencing approach was then used to correct non-stationary time series data. Seasonality was also assessed by conducting the Durbin Watson test and adjusted for using differencing technique whenever applicable.

A number of additional analyses were performed to ensure that the model was best fit, including descriptive statistics and scatter plots of the time series to identify any underlying trends, seasonal patterns and outliers. The pre-implementation period was also checked for linearity and autocorrelation. Likewise, several sensitivity analyses were carried out, including specifying varying interruption points and fitting a regression model for the monthly number of women with PLTC. We also opted to evaluate the impact of the COVID-19 lockdown on the outcome measures in our sensitivity analyses. There is some evidence in the academic literature that the COVID-19 pandemic may be associated with an increased incidence of adverse maternal events, including maternal deaths and severe maternal morbidities [29, 30].

## Reporting

The study is reported as per the TREND statement for improving the reporting quality of nonrandomized evaluations of interventions (Additional file 5).

## Results

In this study, we reviewed medical records of 17,342 women who delivered in the hospital or admitted to the hospital for obstetric emergency reasons. Overall, 4365 women with PLTC were identified during the entire study period. The proportion of mothers with PLTC who experienced SMO (13.39% pre-implementation vs. 4.53% post-implementation; *p* < 0.001), delayed care (59.06% pre-implementation vs. 50.84% post-implementation; *p* < 0.001) and substandard care (29.96% pre-implementation vs. 24.38% post-implementation; *p* < 0.001) was significantly higher in the pre-implementation period compared to the post-implementation period. SMO was the outcome measure that showed the largest difference in proportion between the two study periods. Substandard care, on the other hand, showed the lowest reduction in proportion in the post-implementation period compared to the pre-implementation (Table 1).

**Table 1:** Comparison of primary outcome measures between pre-implementation and post-implementation periods

Outcome variables	Pre-implementation count (% of PLTC)	Post-implementation count (% of PLTC)	Overall count (% of PLTC)	*p*-value *
SMO	345 (13.39)	81 (4.53)	426 (9.76)	< 0.0001
Delayed care	1536 (59.60)	909 (50.84)	2445 (56.01)	< 0.0001
substandard care	772 (29.96)	436 (24.38)	1208 (27.67)	< 0.0001
Total PLTC	2577	1788	4365	

* *prtest* for the equality of the proportion of outcome measures between pre- and post-implementation periods

### Changes in the proportion of SMO cases

**Fig. 1:**
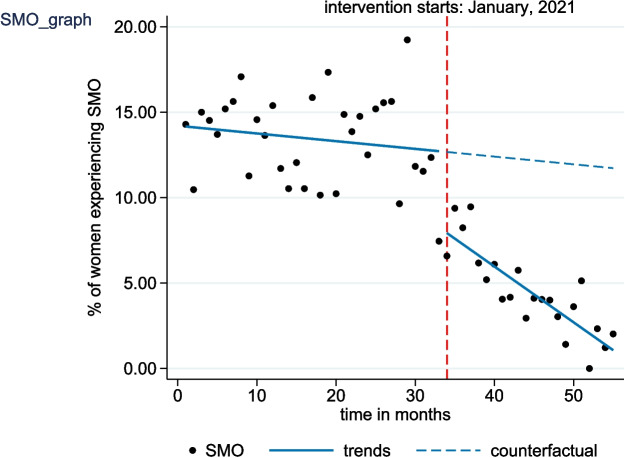
Interrupted time series analysis evaluating the effect of the NMCR cycle on the monthly proportion of severe maternal outcome (SMO) cases

Table 2 presents the results of the regression coefficient of all parameters for the three outcome measures estimated using the interrupted time series analysis. As shown in the table, the starting level of the percentage of SMO among women with PLTC was estimated at 14.16% (95% CI: 12.74 to 15.58; *p* < 0.001). The results also showed that the proportion of SMO cases decreased by 0.05 percentage points (95% CI: − 0.14 to 0.05; *p* = 0.337) monthly before the introduction of the intervention, even though the reduction was statistically insignificant. During the first month of the post-implementation period, the percentage of women with SMO significantly decreased by 4.75% (95% CI: − 6.95 to − 2.54; *p* < 0.001). The observed immediate drop in level was followed by a significant decrease in the monthly trend of the SMO rate (relative to the pre-implementation trend) of 0.28 percentage points (95% CI: − 0.37 to − 0.14; *p* < 0.001) per month. The regression table further shows that the proportion of women with SMO decreased by 0.36 percentage points (95% CI: − 0.60 to − 0.12; *p* < 0.001) per month in the post-implementation period (Fig. 1).

**Table 2:** Changes in the level and trend of primary outcome measures following the introduction of NMCR cycle

Outcome measure	Effect	S.E.	*p*-value	95% CI
**Severe maternal outcome (SMO)**				
Pre-implementation level	14.16	0.71	< 0.001	12.74 to 15.58
Pre-implementation trend	−0.05	0.05	0.337	−0.14 to 0.05
Change in level after implementation	−4.75	1.10	< 0.001	−6.95 to −2.54
Change in trend after implementation	−0.28	0.07	< 0.001	−0.42 to − 0.14
Post-implementation trend	−0.36	0.05	< 0.001	−0.60 to − 0.12
**Delayed care**				
Pre-implementation level	60.65	0.46	< 0.001	59.72 to 61.57
Pre-implementation trend	−0.06	0.03	0.041	−0.13 to − 0.01
Change in level after implementation	−6.91	1.15	< 0.001	−9.28 to −4.68
Change in trend after implementation	0.02	0.07	0.805	−0.11 to 0.15
Post-implementation trend	0.05	0.06	0.516	−0.18 to 0.21
**Substandard care**				
Pre-implementation level	29.77	0.38	< 0.001	29.01 to 30.52
Pre-implementation trend	0.01	0.02	0.642	−0.03 to 0.05
Change in level after implementation	−3.50	0.62	< 0.001	−4.74 to −2.26
Change in trend after implementation	−0.21	0.04	< 0.001	−0.28 to − 0.14
Post-implementation trend	−0.20	0.09	< 0.001	−0.23 to − 0.17

*S.E* standard error, *p*-value cut-off point – 0.05

### Changes in the proportion of delayed care

**Fig. 2:**
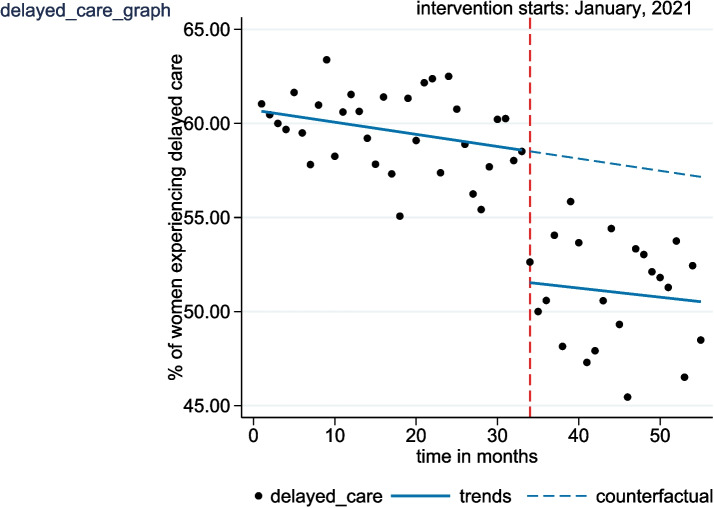
Interrupted time series analyses evaluating the effect of the NMCR cycle on the monthly proportion of delayed care

The regression model fitted for the second outcome measure (delayed care) showed a 7% (95% CI: − 9.28 to − 4.68; *p* < 0.001) decrease in the proportion of delayed care in the first month of the intervention compared to the pre-implementation level. The underlying trend in the pre-implementation period was a slight but statistically significant decrement of 0.06 percentage points (95% CI: − 0.13 to − 0.01; *p* = 0.041) per month. In the post-implementation period there appeared to be a significant increase in the monthly trend of 0.02 percentage points (95% CI: − 0.11 to 0.15; *p* = 0.805) per month relative to the pre-implementation trend. The observed increase in slope did not prove to be statistically significant. The results further indicated that after the initiation of the individual NMCR cycle in January 2021, the trend showed a non-significant monthly increase at a rate of 0.05 percentage points (95% CI: − 0.18 to 0.21; *p* = 0.516) per month (Table 2 and Fig. 2).

### Changes in the proportion of substandard care

**Fig. 3:**
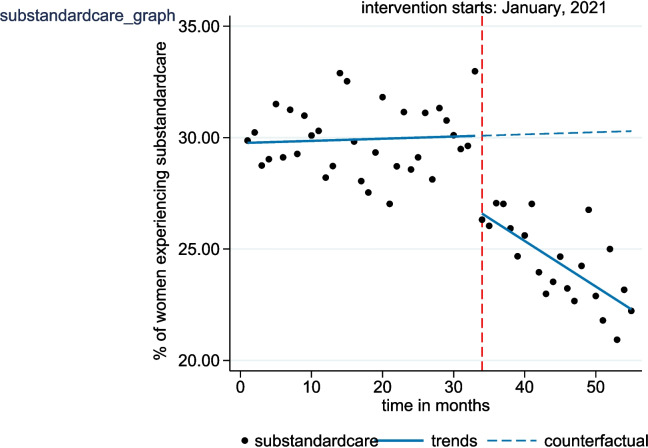
Interrupted time series analysis evaluating the effect of the NMCR cycle on the monthly proportion of substandard care

Changes in the level and trend of the proportion of women with PLTC who received substandard care following the introduction of the individual NMCR cycle are presented in Table 2 and Fig. 3. In the first month of the post-implementation period, a statistically significant decrease in level equivalent to 3.50% (95% CI: − 4.74 to − 2.26; *p* < 0.001) was observed. Similarly, there was a marked decline in the slope of the time series following the intervention. In the pre-implementation period, the trend was flat with insignificant month-to-month increase of 0.01 percentage points (95% CI: − 0.03 to 0.05; *p* = 0.642). After the intervention, however, this trend significantly dropped by 0.21 percentage points (95% CI: − 0.28 to − 0.14; *p* < 0.001) per month relative to pre-implementation trend. Consequently, after the initiation of the individual NMCR cycle, the time series decreased at a rate of 0.20 percentage points (95% CI: − 0.23 to − 0.17; *p* < 0.001) monthly.

## Sensitivity analyses

To ensure the robustness of the final regression model, we carried out a number of sensitivity analyses in this study. First, we fitted an interrupted time series model to the monthly number of women with PLTC to look for any sign of abrupt or gradual change in the monthly count. The fitted model failed to generate any statistically significant change in the level or trend of PLTC count (see Supplementary table S1 in Additional file 3), which can be taken as an important indication of the absence of external factors (occurring alongside the intervention) that affect the monthly number of women who require emergency obstetric interventions. Moreover, additional regression models were fitted for all outcome measures using the data collected on absolute counts instead of computed proportions. The findings were concordant with those obtained from the main analysis in terms of magnitude, direction, and statistical significance (see Supplementary table S2 in Additional file 3). We also fitted another regression model by moving the intervention point to April 2020 (the time when the lockdown was officially declared in Eritrea). Nevertheless, the results did not show any change in level for all outcome measures following the announcement of the lockdown in the country (see Supplementary table S3 in Additional file 3).

## Discussion

In this study, we used an interrupted time series design to investigate the impact of the facility-based individual NMCR cycle in improving maternal outcome and the quality of emergency obstetric care. Our hypothesis was that conducting routine individual NMCR, following the recommendations outlined in the WHO manual, would result in improved quality of emergency obstetric care and favorable maternal outcome in Keren hospital. Concordant with our hypothesis, we observed marked improvements in the outcome measures subsequent to the implementation of the intervention. SMO and substandard care showed a significant drop in level and slope in the regression model following the initiation of the individual NMCR cycle, while delayed care showed a significant change in level only.

In this study, the process indicators proposed in the WHO MNM tool were used to assess whether the individual NMCR cycle improved compliance with evidence-based standards of care. The WHO MNM approach has been previously recommended as a useful quality assessment tool [22]. It has been used to measure the coverage of key process indicators of obstetric care in a number of previous studies [31–33]. Our findings revealed that the initiation of the review cycle resulted in a significant drop in the level and trend of the proportion of women who received substandard care. We attribute this reduction to the critical analysis of the appropriateness of care based on available guidelines and protocols during the monthly sessions of individual NMCR [19]. Here, participants carefully scrutinize the different factors that compromise the quality of emergency obstetric care and try to get to the root cause by repeatedly asking the question “why, but why?” Furthermore, the regular preparation of SMART (i.e. specific, measureable, achievable, realistic and time-bound) recommendations and continuous follow-up of their implementation ensures that improvements made in standards of care are sustained over a long period. Although not directly comparable to ours, previous studies also reported similar findings where obstetric review resulted in improved compliance with agreed standards of care for several life-threatening obstetric complications such as obstructed labor [12, 13], obstetric hemorrhage [14], uterine rupture [16] and severe pre-eclampsia [18]. A study by Lumala et al. [14], for instance, reported that obstetric review improved adherence to seven of 10 predetermined standards of care for ecampsia. Similarly, a study from Tanzania [12] stated that the percentage of women who received standard management of care for obstructed labor increased significantly in the post-audit period.

Timeliness of care is another important dimension of quality of care that should be an integral part of any quality improvement effort [6]. In this study, the proportion of women who received delayed care showed the largest percentage point reduction in the post-implementation period compared to other outcome measures. This finding is most likely due to the meticulous and rigorous analysis of delayed care during the “door-to-door” analysis step of the individual NMCR session [19]. During this step of the NMCR session, the participants thoroughly discuss the causes of all delays that occurred from the time of the mother’s admission to her discharge, and suggest feasible solutions to remedy them. The multidisciplinary nature of the review cycle also fosters communication among staff, possibly contributing to the reduction of delays that occur due to poor communication. Consistent with our findings, a number of studies in the literature also reported improvements in the timeliness of critical interventions subsequent to initiation of near-miss case review including time from admission to specialist review [18, 34], time from indication of caesarean section to delivery [12] and time from diagnosis to treatment [18]. However, it should be noted that delayed care was the only outcome measure that failed to show a significant downward trend during the post-implementation period in this study. Furthermore, the percentage of women who experienced a third delay remained unreasonably high in the months following the introduction of the individual NMCR cycle. Shortage of senior obstetricians and frequent stock outs of important medical supplies and equipment in the hospital [23] are the most plausible explanations for this finding. Such factors are beyond the control of the facility and are less likely to be improved through quality improvement initiatives from hospital members.

The proportion of women with PLTC who experienced SMO was the third primary outcome measure considered for this study. Our findings indicate a significant drop in the SMO proportion following the introduction of the review cycle. Since improvements in the process and timeliness of care are usually expected to lead to better maternal outcome, this finding is most likely due to the observed improvements in quality of care. Even though it is difficult to directly compare these findings to those reported in previous studies due to differences in study design and outcome measures, the results from previous similar studies are generally in line with ours. Most importantly, a recently conducted systematic review reported a 23% reduction in maternal mortality after the implementation of the individual NMCR cycle using a meta-analysis of data pooled from eight studies [11]. Another study from Malawi reported that the quarterly rate of maternal mortality and severe acute maternal morbidity monotonically decreased following the implementation of the obstetric review cycle compared to the baseline rate [35].

### Strengths and limitations

The fact that we used the Donabedian model of quality assessment [26] to measure the impact of the individual NMCR cycle is the main strength of this study. It is difficult to define and operationalize quality of care, let alone decide on how to measure it. However, the model proposed by Donabedian offers the most comprehensive approach to quality assessment since it focuses on the three most important dimensions of care (i.e., structure, process and outcome). This study also benefitted from using standard indicators of process and outcome of care proposed by the WHO [25]. Although these standards do not give an exhaustive list of all possible indicators of quality of care, they allow objective assessment of the most fundamental aspects of emergency obstetric care and maternal outcome. The fact that we used an interrupted time series design to evaluate the effectiveness of our intervention is another positive attribute of this study that merits discussion. Prior evidence on the effectiveness of near miss audits was mainly generated using study designs prone to high risk of bias (mainly uncontrolled before-after design) [9, 11]. Interrupted time series studies are among the most robust observational study designs and can address various threats to internal validity including regression to the mean, secular trends, and unmeasured confounders [36, 37]. Well-designed interrupted time series studies can generate evidence concordant with randomized controlled trials [38], and properly constructed interrupted time series graphs are effective means of communicating results to people with limited technical knowledge [39].

However, our study has some limitations. First, we were not able to measure the magnitude of structural improvement in quality of care associated with the intervention since structural elements of care are not routinely collected in medical registers. Second, our study failed to assess whether the individual NMCR cycle resulted in better care from the mothers’ perspective. The data we collected were secondary data from medical registers, preventing us from directly assessing changes in mothers’ experience of care resulting from the intervention. Third, our study design (single group interrupted time series design) cannot exclude factors occurring concomitantly with the intervention that can affect the observed changes in the outcome measures [40]. However, we took all reasonable precautions to rule out interventions aimed at the outcomes of interest that come into force at or around the time of the individual NMCR implementation. In addition, we carried out several sensitivity analyses based on different scenarios to ensure the robustness of the final model. Finally, this study was conducted in a regional referral maternity hospital that predominantly admits women who need sophisticated emergency obstetric care. To that end, the results from this study may not necessarily be generalizable to all hospitals providing emergency obstetric care, and therefore, our findings should be interpreted with caution in light of this limitation.

### Practice recommendations

To our knowledge, this is the first study to evaluate improvements in quality of emergency obstetric care and maternal outcome associated with the implementation of the individual NMCR cycle developed by the WHO European region. Based on our findings, we strongly recommend its adaptation for use in emergency obstetric facilities in developing countries. One of the most important attributes of the WHO individual NMCR cycle is that only a few cases are evaluated in each session using a qualitative approach [19]. This helps the audit team ensure high quality of the review process and allows highly individualized assessment of care that can easily be tailored to the characteristics of each case. In addition, conducting the individual NMCR cycle addresses several limitations inherent in the traditional methods of obstetric review. First, unlike the criterion-based audit, the individual NMCR does not rely on a narrow list of predefined, explicit standards of care developed for specific categories of cases; therefore, it readily lends itself to variability among cases within and between categories. Second, a few cases are discussed on a regular basis in the individual NMCR cycle, which increases the chances of its sustainability, especially in the context of limited financial and professional resources. Third, since it encourages self-criticism and exchange of views among health professionals, it has the potential to reveal pitfalls in quality of emergency obstetric care that are not usually reported in medical records.

### Future research and policy implications

Despite the insight this study provides into the effect of the individual NMCR cycle in improving quality of emergency obstetric care and maternal outcome, there remains a need for further investigation. Additional research is required with special consideration to address the main limitations identified in this study. Further investigations are desirable to establish whether the individual NMCR cycle leads to better patient judgment of care using suitable study designs, preferably qualitative or mixed methods study designs. In addition, it is important to assess whether the impact of the intervention can be further extended by introducing it along with other quality improvement interventions. Indeed, prior evidence indicates that audits tend to have higher effect when provided as part of a multifaceted intervention than when provided alone [10]. Additional research is also recommended to assess the feasibility of introducing the individual NMCR cycle at the national level and to identify the potential barriers and enablers associated with the implementation process.

## Conclusion

Taken together, our intervention resulted in significant improvements in quality of emergency obstetric care and maternal outcome among women with PLTC. Although the intended beneficiaries of the WHO individual NMCR manual are countries from the WHO European region, our study demonstrates that it can be equally applicable in obstetric facilities of developing countries. The intervention also bears great potential for scaling up, as the WHO manual contains a systematic guide on how to implement the individual NMCR cycle at the national level.

### Supplementary Information

**Additional file 1:.** Aggregate data supporting the findings of this study.

**Additional file 2:.** Operational definition of all outcome variables and the methods used to calculate aggregate outcome measures.

**Additional file 3:.** Results of sensitivity analyses.

**Additional file 4:.** Completed TIDieR template.

**Additional file 5:.** Completed TREND checklist.

## Data Availability

All data generated or analyzed during this study are included in this manuscript and as supplementary files.

## Abbreviations

MNM: Maternal near miss
NMCR: Near miss case review
PLTC: Potentially life-threatening condition
SMO: Severe maternal outcome

## References

[1] Hasan MM, Magalhaes RJS, Fatima Y, Ahmed S, Mamun AA (2021). Levels, trends, and inequalities in using institutional delivery services in low- and middle-income countries: a stratified analysis by facility type. Glob Health Sci Pract.

[2] Ng M, Misra A, Diwan V, Agnani M, Levin-Rector A, Costa AD (2014). An assessment of the impact of the JSY cash transfer program on maternal mortality reduction in Madhya Pradesh, India. Glob Health Action.

[3] Bhandari TR, Dangal G (2014). Emergency obstetric care: strategy for reducing maternal mortality in developing countries. Nepal J Obstet Gynecol.

[4] United Nations. Global strategy for women’s and children’s health. New York; 2010. Available from: https://www.ohchr.org/sites/default/files/Documents/Issues/Women/WRGS/Health/GlobalStrategy.pdf. Accessed 11 March 2023

[5] Chou VB, Walker N, Kanyangarara M (2019). Estimating the global impact of poor quality of care on maternal and neonatal outcomes in 81 low- and middle-income countries: a modeling study. PLoS Med.

[6] World Health Organization. Standards for improving quality of maternal and newborn care in health facilities. Geneva; 2016. Available from: https://cdn.who.int/media/docs/default-source/mca-documents/qoc/quality-of-care/standards-for-improving-quality-of-maternal-and-newborn-care-in-health-facilities.pdf. Accessed 23 March 2023.

[7] World Health Organization. Beyond the numbers: reviewing maternal deaths and complications to make pregnancy safer. Geneva; 2004. Available from: https://apps.who.int/iris/handle/10665/42984. Accessed 12 March 2023.

[8] Ronsmans C. What is the evidence for the role of audits to improve the quality of obstetric care. Safe motherhood strategies: a review of the Evidence 2001.

[9] Kongnyuy EJ, Kabore A, Tebeu PM (2009). Clinical audit to improve obstetric practice: what is the evidence?. Clin Audit.

[10] Ivers N, Jamtvedt G, Flottorp S, Young JM, Odgaard-Jensen J, French SD (2012). Audit and feedback: effects on professional practice and healthcare outcomes. Cochrane Database Syst Rev.

[11] Lazzerini M, Richardson S, Ciardelli V, Erenbourg A (2018). Effectiveness of the facility-based maternal near-miss case reviews in improving maternal and newborn quality of care in low-income and middle-income countries: a systematic review. BMJ Open.

[12] Mgaya AH, Kidanto HL, Nystrom L, Essén B (2016). Improving standards of Care in Obstructed Labour: a criteria-based audit at a referral Hospital in a low-Resource Setting in Tanzania. PLoS One.

[13] Kayiga H, Ajeani J, Kiondo P, Kaye DK (2016). Improving the quality of obstetric care for women with obstructed labour in the national referral hospital in Uganda: lessons learnt from criteria based audit. BMC Pregnancy Childb..

[14] Lumala A, Sekweyama P, Abaasa A, Lwanga H, Byaruhanga R (2017). Assessment of quality of care among in-patients with postpartum haemorrhage and severe pre-eclampsia at st. Francis hospital nsambya: a criteria-based audit. BMC Pregnancy Childb..

[15] Nsangamay T, Mash R (2019). How to improve the quality of care for women with postpartum haemorrhage at Onandjokwe hospital, Namibia: quality improvement study. BMC Pregnancy Childb..

[16] Van den Akker T, Mwagomba B, Irlam J, van Roosmalen J (2009). Using audits to reduce the incidence of uterine rupture in a Malawian district hospital. Int J Gynecol Obstet.

[17] Browne JL, van Nievelt SW, Srofenyoh EK, Grobbee DE, Klipstein-Grobusch K (2015). Criteria based audit of quality of care to women with severe pre-eclampsia and eclampsia in a referral Hospital in Accra, Ghana. PLoS One.

[18] Weeks AD, Alia G, Ononge S, Otolorin EO, Mirembe FM (2005). A criteria-based audit of the management of severe pre-eclampsia in Kampala, Uganda. Int J Gynecol Obstet.

[19] World Health Organization, Regional Office for Europe. Conducting a maternal near-miss case review cycle at the hospital level: manual with practical tools. Copenhagen, Denmark; 2016. Available from: https://www.qualityofcarenetwork.org/sites/default/files/2019-07/NMCR-manual-en.pdf. Accessed 12 March 2023.

[20] Lazzerini M, Ciuch M, Rusconi S, Covi B (2018). Facilitators and barriers to the effective implementation of the individual maternal near-miss case reviews in low/middle-income countries: a systematic review of qualitative studies. BMJ Open.

[21] Filippi V, Ronsmans C, Gohou V, Goufodji S, Lardi M, Sahel A (2004). Maternity wards or emergency obstetric rooms? Incidence of near-miss events in African hospitals. Acta Obstet Gynecol Scand.

[22] Tuncalp O, Souza JP (2014). Maternal near-miss audits to improve quality of care. BJOG..

[23] Zewde HK (2022). Quality and timeliness of emergency obstetric care and its association with maternal outcome in Keren Hospital, Eritrea. Sci Rep.

[24] Hoffmann TC, Glasziou PP, Boutron I, Milne R, Perera R, Moher D (2014). Better reporting of interventions: template for intervention description and replication (TIDieR) checklist and guide. BMJ..

[25] World Health Organization. Evaluating the quality of care for severe pregnancy complications: The WHO near-miss approach for maternal health. Geneva; 2011. Available from: https://apps.who.int/iris/bitstream/handle/10665/44692/9789241502221_eng.pdf;jsessionid=93B9D102E9029688494EABAF54ECEA87?sequence=1. Accessed 20 March 2023.

[26] Donabedian A (1988). The quality of care: how can it be assessed?. JAMA..

[27] Edson W, Burkhalter B, Harvey S, Boucar M, Djibrina S (2006). Safe motherhood studies-timeliness of in-hospital care for treating obstetric emergencies: results from Benin, Ecuador, Jamaica, and Rwanda. Operations research results.

[28] Linden A (2015). Conducting interrupted time series analysis for single and multiple group comparisons. Stata J.

[29] Chmielewska B, Barratt I, Townsend R, Kalafat E, van der Meulen J, Gurol-Urganci I (2021). Effects of the COVID-19 pandemic on maternal and perinatal outcomes: a systematic review and meta-analysis. Lancet Glob Health.

[30] Marchand G, Patil AS, Masoud AT, Ware K, King A, Ruther S, et al. Systematic review and meta-analysis of COVID-19 maternal and neonatal clinical features and pregnancy outcomes up to June 3, 2021. AJOG Glob Rep 2022; 2(1):100049. 10.1016/j.xagr.2021.100049. 10.1016/j.xagr.2021.100049 PMC8720679 35005663

[31] Tuncalp O, Hindin MJ, Adu-Bonsaffoh K, Adanu RM (2013). Assessment of maternal near-miss and quality of care in a hospital-based study in Accra, Ghana. Int J Gynaecol Obstet.

[32] Jabir M, Abdul-Salam I, Suheil DM, Al-Hilli W, Abul-Hassan S, Al-Zuheiri A (2013). Maternal near miss and quality of maternal health care in Baghdad, Iraq. BMC Pregnancy Childb..

[33] Nelissen E, Mduma E, Ersdal HL, Evjen-Olsen B, van Roosmalen J, Stekelenburg J (2013). Maternal near miss and mortality in a rural referral hospital in northern Tanzania: cross-sectional study. BMC Pregnancy Childb..

[34] Kidanto HL, Wangwe P, Kilewo CD, Nystrom L, Lindmark G (2012). Improved quality of management of eclampsia patients through criteria based audit at Muhimbili National Hospital, Dar Es Salaam, Tanzania. Bridging the quality gap. BMC Pregnancy Childb..

[35] van den Akker T, van Rhenen J, Mwagomba B, Lommerse K, Vinkhumbo S, van Roosmalen J (2011). Reduction of severe acute maternal morbidity and maternal mortality in Thyolo District, Malawi: the impact of obstetric audit. PLoS One.

[36] Penfold RB, Zhang F (2013). Use of interrupted time series analysis in evaluating health care quality improvements. Acad Pediatr.

[37] Wagner AK, Soumerai SB, Zhang F, Ross-Degnan D (2002). Segmented regression analysis of interrupted time series studies in medication use research. J Clin Pharm Ther.

[38] Fretheim A, Soumerai SB, Zhang F, Oxman AD, Ross-Degnan D (2013). Interrupted time-series analysis yielded an effect estimate concordant with the cluster-randomized controlled trial result. J Clin Epidemiol.

[39] Turner SL, Karahalios A, Forbes AB, Taljaard M, Grimshaw JM, Korevaar E (2020). Creating effective interrupted time series graphs: review and recommendations. Res Syn Meth.

[40] Svoronos T (2016). Evaluating Health Interventions Over Time: Empirical Tests of the Validity of the Single Interrupted Time Series Design. Doctoral dissertation.

