# Supplementary material for: Using the WHO individual near miss case review (NMCR) cycle to improve quality of emergency obstetric care and maternal outcome in Keren hospital, Eritrea: an interrupted time series analysis
Source: BMC Pregnancy Childbirth. 2024 Apr 11;24:266. doi: 10.1186/s12884-024-06482-3 (PMC11010365; doi:10.1186/s12884-024-06482-3)
Supplement: Supplementary file 2 — Additional file 2:. Operational definition of all outcome variables and the methods used to calculate aggregate outcome measures. [file 12884_2024_6482_MOESM2_ESM.docx]

**Operational definition of outcome variables and aggregate outcome measures used in this study**

1. **Operational definition of variables related to outcome of care**

**Maternal near miss (MNM):** refers to a woman who nearly died but survived a complication that occurred during pregnancy, childbirth or within 42 days of termination of pregnancy.

**Maternal death (MD):** is the death a woman while pregnant or within 42 days of termination of pregnancy or its management, but not from accidental or incidental causes.

**Severe maternal outcome (SMO):** refers to a life-threatening condition (i.e. organ dysfunction), including all maternal deaths and maternal near miss cases.

**Potentially life-threatening conditions (PLTC):** refers to five life threatening obstetric conditions i.e. severe post-partum hemorrhage, severe pre-eclampsia, eclampsia, sepsis (severe systemic infection), and uterine rupture.

1. **Aggregate measure for outcome of care**

$\%\boldsymbol{of women wi}\boldsymbol{th SMO=}\frac{\boldsymbol{\# of women who experienced SMO}}{\boldsymbol{\# of women with PLTC}}$ *** 100%**

1. **Operational definition of variables related to timeliness of care**

**Delay in professional evaluation**: delay in professional evaluation after the mother’s admission to hospital.

**Delay in making diagnosis**: delay in making the correct diagnosis once the mother is attended by a professional

**Delay in definitive treatment**: delay in giving the woman the definitive treatment and/or management after making the right diagnosis.

**N.B**- the decision whether there is any delay in the above categories rests on the judgment of the obstetricians who collected data for this study.

1. **Aggregate outcome measure for timeliness of care**

$\boldsymbol{\% of delayed care=}\frac{\boldsymbol{\# of women who experienced at least one type of delay}}{\boldsymbol{\# of women with PLTC}}$ *** 100%**

1. **Operation definition of variables related to standards of care**

**Treatment of postpartum hemorrhage (PPH)**: whether a woman diagnosed with PPH received uterotonics.

**Anticonvulsants for eclampsia**: whether a woman diagnosed with eclampsia received Magnesium sulfate or other anticonvulsant.

**Prevention of caesarean section related infection**: whether a woman undergoing caesarean section received prophylactic antibiotic.

**Treatment for sepsis**: whether a woman diagnosed with sepsis received parenteral therapeutic antibiotics.

**Laparotomy for uterine rupture**: whether a woman diagnosed with uterine rupture performed laparotomy within three hours of hospital stay.

1. **Aggregate outcome measure for process of care**

$\boldsymbol{\% of substandard care=}\frac{\boldsymbol{\# of women with atleast one substandard care}}{\boldsymbol{\# of wome}\boldsymbol{n with PLTC}}$ ***100%**
