# Supplementary material for: Using the WHO individual near miss case review (NMCR) cycle to improve quality of emergency obstetric care and maternal outcome in Keren hospital, Eritrea: an interrupted time series analysis
Source: BMC Pregnancy Childbirth. 2024 Apr 11;24:266. doi: 10.1186/s12884-024-06482-3 (PMC11010365; doi:10.1186/s12884-024-06482-3)
Supplement: Supplementary file 3 — Additional file 3:. Results of sensitivity analyses. [file 12884_2024_6482_MOESM3_ESM.docx]

**Supplementary table S1. Changes in the level and trend of monthly PLTC count following the introduction of the NMCR cycle**

| **Parameter** | **Effect** | **S.E.** | **p-value** | **95% CI** |
| --- | --- | --- | --- | --- |
| Pre-intervention level | 75.31 | 2.97 | <0.001 | 69.34 to 81.28 |
| Pre-intervention trend | 0.17 | 0.17 | 0.306 | -0.16 to 0.51 |
| Change in level after intervention | -0.56 | 5.10 | 0.914 | -10.80 to 9.67 |
| Change in trend after intervention | -0.10 | 0.34 | 0.774 | -0.79 to 0.59 |
| Post-intervention trend | 0.07 | 0.31 | 0.861 | -0.87 to 0.96 |

S.E – standard error, p-value cut-off point – 0.05, CI – Confidence Interval

**Supplementary table S2. Changes in the level and trend of the absolute count of outcome measures following the introduction of the NMCR cycle**

| **Outcome measure** | **Effect** | **p-value** | **95% CI** |
| --- | --- | --- | --- |
| **Severe maternal outcome (SMO)** | | | |
| Change in level after intervention | -3.63 | <0.001 | -5.58 to -1.68 |
| Change in trend after intervention | -0.25 | 0.010 | -0.37 to -0.13 |
| **Delayed care** | | | |
| Change in level after intervention | -6.08 | 0.034 | -11.68 to -0.49 |
| Change in trend after intervention | -0.06 | 0.719 | -0.40 to 0.27 |
| **Substandard care** | | | |
| Change in level after intervention | -3.05 | 0.067 | -6.33 to -0.23 |
| Change in trend after intervention | -0.22 | 0.020 | -0.40 to -0.04 |

p-value cut-off point – 0.05, CI – Confidence Interval

**Supplementary table S3. Changes in the level of primary outcome measures following the declaration of COVID-19 related lockdown in Eritrea**

| **Outcome measure** | **Effect** | **p-value** | **95% CI** |
| --- | --- | --- | --- |
| **Severe maternal outcome (SMO)** | | | |
| Change in level after intervention | 1.19 | 0.269 | -0.95 to 3.32 |
| **Delayed care** | | | |
| Change in level after intervention | -1.94 | 0.173 | -4.77 to 0.88 |
| **Substandard care** | | | |
| Change in level after intervention | 1.30 | 0.128 | -0.40 to 3.00 |

p-value cut-off point – 0.05, CI – Confidence Interval
