# Supplementary material for: Using the WHO individual near miss case review (NMCR) cycle to improve quality of emergency obstetric care and maternal outcome in Keren hospital, Eritrea: an interrupted time series analysis
Source: BMC Pregnancy Childbirth. 2024 Apr 11;24:266. doi: 10.1186/s12884-024-06482-3 (PMC11010365; doi:10.1186/s12884-024-06482-3)
Supplement: Supplementary file 4 — Additional file 4:. Completed TIDieR template. [file 12884_2024_6482_MOESM4_ESM.docx]

**Completed TIDieR template for NMCR cycle intervention**

| 1. **Brief name** | WHO individual near-miss case review (NMCR) cycle at hospital level |
| --- | --- |
| 1. **Why** | The main objective of this intervention is to improve the quality of maternal care and outcome using systematic review of care against evidence-based criteria. The intervention (the WHO NMCR cycle) is unique in its approach from commonly used methods of criterion based audit for a number of reasons:   - It provides detailed guidance on how to evaluate the quality of care from the woman’s perspective. - It encourages active involvement of the staff who provided care for the woman rather than external auditors which makes solutions more likely to be proposed, agreed, and implemented - It emphasizes discussion among staff members who participated in providing care for the woman to expose a number of deficiencies and concerns in quality of care rather than solely relying on information collected from medical records - It advocates for pragmatic and individualized assessment of care and does not depend on narrow list of predetermined and inflexible standards of care. - Few cases (one or two) are discussed each month and little resource is required to organize and run the review increasing the chances of its sustainability |
| 1. **What (material)** | 1. The process of conducting NMCR cycle was planned and implemented in accordance with a manual prepared by the WHO regional office for Europe [1]. The manual contains a detailed instruction on how to prepare for each session of NMCR, the different steps that need to be followed in each session, and how to plan and implement recommended actions after each session 2. Required medical records of the case(s) under discussion are retrieved by the facilitator before the commencement of each session to aid in the process of review 3. A summary of the case interview with the woman or her family 4. A blank template provided in the manual (annex 10) for case analysis and recommendations 5. Standard textbooks and national guidelines which are used as a reference of standards of care. Scientific papers and articles from reputed publishers are also used occasionally as needed 6. A logbook to take note of the discussions and recommendations made during each session |
| 1. **What (Procedures)** | - Before taking the initiative to implement the NMCR cycle at hospital level, on-the-job training was given to all staff of the hospital who are likely to participate in the management of obstetric emergencies. Participants of the training were from diverse areas of practice (health assistants, nurse midwives, laboratory professionals, pharmacists etc.). All materials for the training were taken from the NMCR manual. The training focused on the purpose of conducting routine NMCR and the steps that need to be followed to ensure the quality of the review process. - Meetings are organized monthly and one or two cases of near miss are usually discussed in each session. All women admitted in a given month who fulfil the WHO maternal near-miss criteria are considered eligible. Near miss cases are then selected based on their educational value and the facilitator always makes sure that the cases represent a different condition/complication (and thus different learning opportunity) from those discussed in earlier review sessions. - The head of the maternity unit of the hospital coordinates activities of NMCR and is the one designated as coordinator. The coordinator assigns a facilitator for the next session of NMCR at the end of each session. For the first session of the review the coordinator assumes the role of facilitator as well. The coordinator and facilitator guide the review process according to their roles and responsibilities as stated in the manual. - A set of ground rules and code of conduct were developed and posted on the room where regular meetings are held. They were adapted from the manual with little modifications based on the recommendations from the participants. - The person who took the responsibility to interview the woman whose case is selected for discussion prepares a summary of the case interview and presents it in the meeting - Each session of NMCR is carried out following the steps clearly stipulated in the manual. The facilitator is the one who ensures the steps are strictly adhered to. |
| 1. **Who provided** | - *On-the-job training providers*: the principal researcher along with two regional authorities of maternal and child health were responsible for the training. These individuals were deemed to be sufficiently qualified for the task as they have years of experience on the implementation of quality improvement projects related to maternal and child health. - *NMCR coordinator*: the head of the maternity unit of the hospital is the one who takes the responsibility of coordinating NMCR activities in the hospital. She is the one who oversees and organizes emergency obstetric care activities in the hospital, and therefore she can easily embrace the responsibility of coordinating the review process. - *NMCR facilitator*: new facilitator is appointed for each session of NMCR. Any health personnel who actively engage in caring obstetric emergencies can be a facilitator. The coordinator is the one who appoints the facilitator for each session. - *Quality of NMCR cycle assessors*: the principal researcher and the two regional authorities of maternal and child health, who provided the initial training, are also responsible for assessing the quality of review cycle and providing timely and appropriate feedback |
| 1. **How** | - *Training*: the training was given using power point slides prepared from the manual. Presentations were given using LCD projector. The training was hands-on. Participants were encouraged to put their ideas and concerns forward freely and to express their doubts without any hesitation. At the end of the training participants were divided into groups with each group represented by different professions. All groups underwent a simulation exercise to familiarize themselves with the process of NMCR cycle. - *NMCR session*: the coordinator and the facilitator are charged with overseeing the review process. Once the case summary is presented, the facilitator invites the participants to provide additional suggestions ensuring that everybody’s voice is heard |
| 1. **Where** | All activities of the NMCR (including the training) were held in Keren hospital’s conference room |
| 1. **When and how much** | - Since its introduction in January 2021 the NMCR was established as a routine practice in Keren hospital. Sessions are scheduled monthly and the coordinator is responsible for setting the date and time of review sessions. Sessions are typically scheduled at the beginning of each month. Each session usually takes one hour to one hour and half. - Most of the time one case is discussed in each session. Occasionally, if the facilitator finds more than one case that have important educational value, two cases can be presented in a meeting. However, the facilitator cannot bring more than two cases for discussion in a single session. |
| 1. **tailoring** | N/A |
| 1. **modifications** | - Even though the hospital medical director was intended to be among the participants of the NMCR cycle, his presence in the first session of the review seemed to obscure participants from freely participating in the discussion. Therefore, following an agreement reached between the principal researcher and the medical director, it was decided that he stops attending the NMCR monthly sessions - Despite the initial plans to conduct maternal case interview for each case chosen for discussion in every session, it proved difficult when two cases were selected for a single session. Hence, in such circumstances, the facilitator compares the two cases and he/she only interviews the one that he/she feels can create more learning opportunities |
| 1. **How well (planned)** | - Fidelity of the NMCR cycle was assessed using a checklist provided in the manual (annex 16). Following the commencement of the NMCR cycle, external assessors visit the hospital every six months and assess the quality of the NMCR adhering to the principles and instructions stated in the manual. After each assessment, they score the quality of the review cycle using scoring criteria. The methods used to assess the quality of the review cycle are described in detail in the manual. The checklist contains 11 domains and 50 items. Each item is given a score ranging from 0 (totally inappropriate) to 3 (appropriate). An aggregated domain score is calculated for each domain by calculating the mean of the items score in that particular domain. They also prepare feedback using the matrix of recommendations for improving the quality of NMCR cycle provided in the manual (annex 16). |
| 1. **How well (actual)** | - Overall, the quality of the NMCR cycle was good during the first round of quality assessment (2.26). Major problems were noted in the following domains: developing SMART recommendations, following the ground rules and code of conduct, and properly documenting the review session. Immense improvements were observed in the subsequent rounds of quality assessments. Most of the items received a score of 3 and none received a score of lower than two (mean score = 2.87). |

1. World Health Organization, Regional Office for Europe. Conducting a maternal near-miss case review cycle at the hospital level: manual with practical tools. Copenhagen, Denmark; 2016. Available from: <https://www.qualityofcarenetwork.org/sites/default/files/2019-07/NMCR-manual-en.pdf>
